# Dietary Butyrate as a Functional Feed Additive in Aquaculture: A Systematic Review and Meta‐Analysis of Immunological and Antioxidant Responses With Dose–Response Insights

**DOI:** 10.1155/anu/1873426

**Published:** 2026-10-21

**Authors:** Moussa Gouife, Tchouli Noufeu, Yubin Chen, Xiwen Jia, Jiajie Xu

**Affiliations:** ^1^ Microbial Development and Metabolic Engineering Laboratory School of Marine Science Ningbo University Ningbo 315211 China nbu.edu.cn; ^2^ National Advanced School of Maritime and Ocean Science and Technology (ENSTMO) University of Ebolowa Kribi Cameroon; ^3^ National Higher School of Agriculture Fisheries and Veterinary Medicine (ENSAHV) University of Douala Douala Cameroon univ-douala.cm

**Keywords:** antioxidant capacity, butyrate, dose–response modeling, functional feed additive, immune responses, meta‑analysis

## Abstract

ABSTRACT Aquaculture intensification has increased the demand for nutritional strategies that support immune responses and oxidative balance in cultured finfish and crustaceans, thereby reducing disease susceptibility and improving production stability. Butyrate, a short‐chain fatty acid increasingly incorporated into aquafeeds as a functional feed additive, has shown promise in enhancing immune responses and antioxidant defenses, but reported effects vary widely across species, life stages, and supplementation levels. This systematic review and meta‐analysis synthesizes findings from 65 studies (1174 dose–control comparisons) across 23 aquaculture species to assess butyrate’s effects on innate and adaptive immunity, oxidative stress, and antioxidant capacity. Dietary butyrate generally enhanced innate immune responses (*g* = 2.14, 95% CI: 1.66–2.63) and antioxidant enzyme activity (*g* = 1.59, 95% CI: 1.07–2.12), increased IL‐10 (*g* = 0.64), and reduced MDA (*g* = −1.36). Effects on adaptive immunity were positive but more heterogeneous among studies (*g* = 0.98, 95% CI: 0.33–1.63), and pooled effects on pro‐inflammatory cytokines, chemotactic cytokines, and immune signaling pathways were not significant. Trim‐and‐fill adjustment attenuated the pooled estimates for innate immunity (*g* = 1.33) and antioxidant enzymes (*g* = 0.84), indicating possible small‐study effects. In dose–response meta‐regression, dietary inclusion level explained little of the heterogeneity (pseudo‐*R* ^2^ ≤6.3%), and no inclusion range was consistently associated with stronger responses. These findings support butyrate as a promising functional feed additive, while indicating that dose recommendations require species‐specific dose–response trials.

## 1. Introduction

Aquaculture has become the fastest‐growing food production sector globally, expanding from less than 10% of the world’s fish supply in the 1970s to more than half of all aquatic foods consumed today [1]. This rapid growth is largely driven by increasing global demand for aquatic protein and the pressure to supply sustainable, high‐quality food for a growing human population [2]. However, intensifying aquaculture systems have significantly increased risks to the health and welfare of cultured species. As production systems become more crowded and environmental stressors intensify, the resilience of farmed species, particularly fish and crustaceans, has been increasingly compromised [3, 4].

Infectious diseases remain a primary challenge to sustainable aquaculture, with global economic losses from disease outbreaks estimated at USD 6–10 billion annually [5]. Consequently, concern is growing about the increasing reliance on antibiotics and other chemotherapeutics to manage disease, especially given rising issues related to antimicrobial resistance, environmental contamination, and food safety [6].

Oxidative stress represents a critical challenge in intensive aquaculture [7–10]. Cultured fish and crustaceans are continuously exposed to environmental stressors, including high stocking densities, fluctuating water quality, handling procedures, and repeated pathogen exposure, which increase reactive oxygen species (ROS) production beyond the capacity of endogenous antioxidant systems [11–13]. Elevated ROS can damage cellular macromolecules, promote chronic inflammation, impair immune responses, and reduce growth performance [14–17]. The antioxidant defense system, which includes both enzymatic (superoxide dismutase, catalase, glutathione peroxidase (Gpx)) and nonenzymatic (glutathione (GSH), total antioxidant capacity) components, plays a central role in maintaining redox homeostasis. The close interconnection between immune pathways and oxidative stress regulation suggests that nutritional interventions targeting both systems could effectively enhance the resilience of aquaculture species [8, 18].

Over the past decade, functional feed additives have emerged as key components of precision nutrition strategies in aquaculture, offering promising solutions to improve fish health, enhance disease resistance, and reduce dependence on antibiotics [19–21]. Among these additives, butyrate, a short‐chain fatty acid produced during microbial fermentation, has garnered significant attention for its pleiotropic effects on immune responses, oxidative stress regulation, and overall health in aquatic species [22–25]. Butyrate serves as both an energy source for intestinal epithelial cells and a signaling molecule regulating various immune and metabolic pathways [26–28]. However, in aquatic animals, endogenous butyrate production is often insufficient because gut microbial communities have relatively low fermentative capacity compared with those of terrestrial herbivores, so dietary supplementation is often needed to achieve physiologically relevant concentrations [29–31].

Numerous experimental studies have reported that dietary butyrate supplementation enhances innate immune responses, improves intestinal barrier integrity, modulates cytokine expression, stimulates antioxidant enzyme activities, and enhances resistance to bacterial pathogens in both fish and crustaceans [32–37]. Additional benefits have also been reported in growth performance, feed utilization efficiency, nutrient digestibility, intestinal morphology, and beneficial modulation of gut microbiota composition [38–41]. Despite these promising results, outcomes of butyrate supplementation have been highly variable, depending on species, developmental stage, dietary formulation, butyrate form, and especially dosage, which ranges from 0.05 to 5 g kg^−1^ diet [22, 23, 42]. While some studies report significant physiological benefits at low inclusion levels [43, 44], others observe improvements only at higher doses or find no effects at all [45, 46]. As such, the dose–response relationship for butyrate supplementation remains poorly understood.

In addition to variation in effect sizes, existing studies on butyrate supplementation often suffer from methodological limitations, including small sample sizes, heterogeneous biomarkers, and limited replication. These issues complicate cross‐study comparisons and hinder the identification of generalizable patterns. Despite a growing body of literature, previous reviews have addressed various aspects of butyrate supplementation in aquaculture. However, to our knowledge, no meta‐analysis has yet jointly quantified the immunological and antioxidant effects of dietary butyrate across both finfish and crustaceans. Such a synthesis is urgently needed to clarify whether butyrate supplementation consistently enhances immune and antioxidant responses and to identify the biological and methodological factors that account for the variability across studies.

Here, we present the first comprehensive meta‐analysis and meta‐regression assessing the effects of dietary butyrate supplementation on immuno‐oxidative responses in aquaculture species. Specifically, we aim to (1) quantify the effects of butyrate across key physiological domains, including innate immunity, adaptive immunity, inflammatory signaling, antioxidant defenses, and oxidative stress biomarkers; (2) explore the relationship between the dietary inclusion level and these responses; and (3) describe the available evidence and assess the robustness of the pooled estimates to dependence among effect sizes, dominant species, and small‐study effects. By synthesizing data from over a decade of experimental research, this study clarifies butyrate’s role as a functional feed additive in aquaculture nutrition and provides evidence‐based guidance for feed formulation, health management, and sustainable production in intensive aquaculture systems.

## 2. Methods

This systematic review and meta‐analysis was conducted and is reported in accordance with the PRISMA 2020 guidelines [47]; the completed PRISMA 2020 checklist is provided as Supporting Information 1.

### 2.1. Study Selection and Literature Search

To assess the effects of dietary butyrate supplementation on immuno‐oxidative responses in aquaculture species, we conducted a comprehensive systematic literature search in October 2025 using Web of Science, Scopus, and AGRIS. The search captured studies investigating butyrate as a feed additive and its effects on immune responses and oxidative stress. Search terms included (“butyrate” OR “butyric acid” OR “SCFA” OR “short‐chain fatty acid”) AND (“aquaculture” OR “fish” OR “shellfish” OR “crustaceans” OR “shrimp” OR “prawn”) AND (“immune response” OR “oxidative stress” OR “antioxidant enzymes” OR “immune modulation”). Boolean operators and wildcard symbols were employed to maximize retrieval while minimizing irrelevant studies. Additionally, backward and forward citation tracking was performed to identify additional relevant studies that may have been missed in the initial search [48].

The inclusion criteria were (i) studies involving fish and shellfish species cultured in aquaculture systems; (ii) studies that administered butyrate in any form and at any level, predominantly through dietary supplementation; and (iii) studies reporting quantitative outcomes related to immune and oxidative biomarkers, such as antioxidant enzyme activity, immune gene expression, or oxidative stress indicators. Studies were excluded if they (i) lacked a control group or extractable quantitative data, (ii) were not conducted under aquaculture conditions or focused on terrestrial species, or (iii) were reviews, conference abstracts, or duplicates.

Two reviewers (Moussa Gouife and Tchouli Noufeu) independently screened titles and abstracts, followed by full‐text assessment. Disagreements were resolved by consensus or, when necessary, by a third reviewer (Jiajie Xu). After screening, 65 studies met the inclusion criteria, providing 1174 individual dose–control comparisons from a wide range of species and experimental conditions (Figure 1). We supplemented the initial search with additional relevant studies identified through the reference lists of the selected articles. The complete list of included studies and their bibliographic details is provided in Supporting Information 2: Table S1.

**Figure 1:**
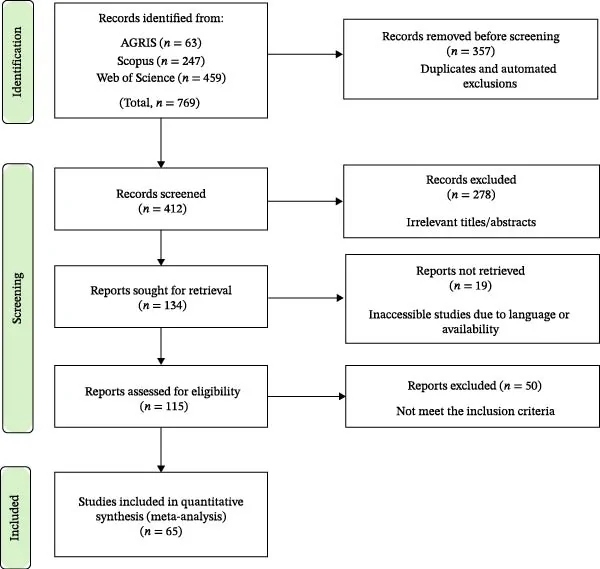
PRISMA flow diagram summarizing the literature search and study selection process for evaluating the effects of dietary butyrate on immune and antioxidant responses in finfish and crustaceans.

### 2.2. Data Extraction

Data extraction was conducted using a standardized template adapted from meta‐analysis guidelines to ensure consistency and reproducibility [49]. Key information extracted from each study included (i) study details (authors, year, journal, study location); (ii) species information (scientific name, taxonomic classification, habitat type, life stage); (iii) experimental conditions (dietary butyrate inclusion levels, formulation, supplementation duration, stocking density, water quality parameters, feeding regime); and (iv) biomarkers and outcome variables (types of immune responses, antioxidant enzymes, oxidative stress markers), along with the corresponding standard deviations or standard errors. We recorded environmental and methodological variables, stocking density, water quality parameters, and feeding regime to characterize each study’s experimental conditions but did not enter them as moderators in the meta‐regression because reporting was inconsistent across included studies. We extracted the form of butyrate supplementation whenever reported. Biomarkers were assigned to outcome categories as follows: innate immunity (lysozyme, complement components, respiratory burst, phagocytosis, myeloperoxidase); adaptive immunity (immunoglobulin M, antimicrobial peptides, lymphocyte indices, antiprotease); anti‐inflammatory cytokines (IL‐10, TGF‐β); pro‐inflammatory cytokines (IL‐1β, IL‐6, TNF‐α); chemotactic cytokines (IL‐8, IFN‐γ); immune signaling pathways (NF‐κB, TLRs, MyD88); antioxidant enzymes (SOD, CAT, GPx); nonenzymatic antioxidants (GSH, T‐AOC); oxidative stress markers (ROS, MDA) (Supporting Information 3: Table S2). For studies reporting multiple inclusion levels, each dose–control comparison was treated as a separate unit of analysis to allow dose–response analyses [49]. Graphical data were extracted using WebPlotDigitizer [50]. Where necessary, authors were contacted to obtain missing data or clarify experimental details.

### 2.3. Statistical Analysis

#### 2.3.1. Effect Size Calculation

The primary outcome metric was the standardized mean difference (Hedges’ *g*), which is appropriate for comparing continuous biological traits such as immune and oxidative markers [51]. Effect sizes were calculated from the reported group means, standard deviations, and sample sizes. In studies comparing multiple treatment groups with a shared control, each contrast was treated as a separate effect size to allow dose–response analyses.

Because multiple effect sizes were derived from the same biological system, the observations cannot be treated as fully independent. Effect sizes were aggregated at the biomarker level within each species before pooling, and all pooled models were additionally refitted as multilevel random‐effects models (rma.mv, metafor) with species as the clustering variable, complemented by cluster‐robust variance estimation (RVE) with Satterthwaite degrees of freedom (clubSandwich) [52]. For species represented by a single study, this clustering is exactly equivalent to the study‐level clustering. For species represented by several studies, the biomarker‐level aggregation step already accounts for between‐study pooling within that species. The variance components (*τ* ^2^) were estimated at the species level and ranged from 0.08 to 1.31 across outcomes. Standard random‐effects models (metagen, meta) were used for the primary pooled estimates and forest plots; multilevel and RVE estimates are reported as sensitivity analyses [49].

#### 2.3.2. Heterogeneity Assessment

Between‐study heterogeneity was evaluated using Cochran’s *Q* statistic and the *I* ^2^ statistic [53], which measures the proportion of total variability due to heterogeneity rather than sampling error. *I* ^2^ values of 25%, 50%, and 75% were interpreted as low, moderate, and high heterogeneity, respectively. The between‐study variance (*τ* ^2^) was estimated using restricted maximum‐likelihood (REML) [54]. Forest plots were generated to visually represent effect sizes and heterogeneity across studies.

#### 2.3.3. Publication Bias

Potential publication bias was assessed using funnel plots, which allow visual inspection of asymmetry that might indicate publication bias toward studies with significant results [55]. The presence of small‐study effects was tested using Egger’s regression test and Begg’s rank correlation test [56]. When asymmetry was detected, the trim‐and‐fill method was employed to estimate potentially missing studies and adjust pooled effect sizes [57]. Egger’s regression and Begg’s rank correlation tests were performed only for outcome categories including at least 10 effect sizes (*k* ≥ 10), as both tests are statistically underpowered below this threshold and prone to false‐negative and false‐positive results.

#### 2.3.4. Meta‐Regression

To explore the relationship between butyrate dosage and physiological outcomes, we used meta‐regression models implemented in metafor [52]. We treated dietary butyrate inclusion level as a continuous moderator. For each outcome category, a mixed‐effects meta‐regression model was fitted with dose as the sole continuous moderator. We computed slope coefficients, 95% confidence intervals, *p*‐values, pseudo‐*R* ^2^ values, and the omnibus test of moderators (Q_model) for each model. Pseudo‐*R* ^2^ represents the proportion of residual heterogeneity accounted for by the moderator. Q_model tests whether the moderator explains a significant amount of variance in the effect sizes.

To assess whether the pooled estimates were driven by the most frequently studied species, we conducted a sensitivity analysis in which we excluded the three dominant species (largemouth bass, Pacific white shrimp, and Nile tilapia) and refitted all pooled models.

#### 2.3.5. Study Quality and Reporting Completeness

A formal randomization and blinding‐based risk‐of‐bias instrument could not be applied, as allocation concealment and blinding of outcome assessment are only rarely reported in aquaculture feeding trials. Instead, methodological quality was appraised through a structured reporting‐completeness assessment covering 10 pre‐specified experimental parameters (replication, stocking density, initial body weight, acclimation period, water temperature, pH, dissolved oxygen, ammonia concentration, experimental duration, and feeding frequency), each scored as reported or not reported for every included study. The 10 parameters were extracted for each study and are reported in Supporting Information 2: Table S1. The certainty of evidence was not assessed using GRADE because the included studies were highly heterogeneous and outcomes were biomarker‐based.

### 2.4. Statistical Software and Packages

All statistical analyses were conducted using R v4.4.1 [58]. Standard random‐effects models and forest plots were performed using the meta package; multilevel models and meta‐regressions using metafor; and RVE using clubSandwich [52]. Supplementary visualizations were created using Python v3.10, while ggplot2 [59] was employed for data visualization. Data from figures were extracted using WebPlotDigitizer [50].

## 3. Results

### 3.1. Evidence Synthesis and Study Characteristics

The systematic literature search identified 65 studies meeting the inclusion criteria for quantitative synthesis (Figure 1), yielding 1174 individual dose–control comparisons. The 50 studies excluded at the full‐text stage did not meet one or more of the eligibility criteria (no control group, no quantitative immune/oxidative outcomes, or not conducted under aquaculture conditions). Research activity has expanded markedly since 2018, with ~85% of studies published in the last 7 years, reflecting growing scientific interest in using functional feed additives to enhance health and resilience in aquaculture (Figure 2).

**Figure 2:**
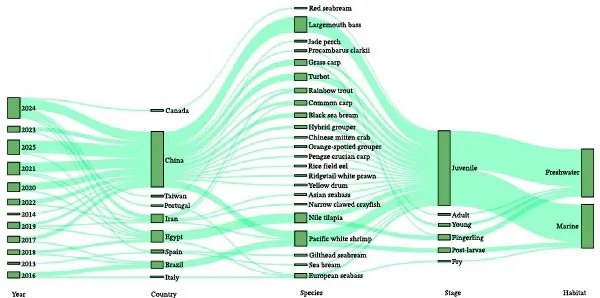
Sankey diagram illustrating the distribution of included studies by publication year, geographic origin, aquatic species, developmental stage, and habitat (*n* = 65).

Geographically, the evidence base was dominated by Asian aquaculture systems (72%), with major contributions from China (62%), Iran (9%), and Taiwan (2%), alongside additional studies from Europe, Africa, and South America. The most frequently investigated species were largemouth bass (*Micropterus salmoides*; *n* = 11 studies, 16.9%) and Pacific white shrimp (*Litopenaeus vannamei*; *n* = 11 studies, 16.9%), followed by Nile tilapia (*Oreochromis niloticus*; *n* = 7 studies, 10.8%), turbot (*Scophthalmus maximus*; *n* = 5 studies), and grass carp (*Ctenopharyngodon idella*; *n* = 4 studies). Eleven species were represented by a single study each, reflecting a marked imbalance in species coverage across the available literature (Figure 2).

Experimental systems covered a broad range of aquaculture environments, with freshwater studies predominating (52%) and marine systems accounting for 48%. Most experiments focused on juvenile developmental stages (80%), reflecting their greater physiological plasticity and economic importance in aquaculture production. Among butyrate supplementation forms, sodium butyrate was the most commonly used formulation (Figure 2).

Dietary inclusion levels showed substantial variation, spanning 0.05–5.0 g kg^−1^ diet, a 100‐fold range. This range allowed dose–response and meta‐regression analyses to be performed across a broad range of inclusion levels. Figure 2 illustrates the distribution of studies by geographic origin, species, developmental stage, and habitat.

Reporting of experimental conditions was generally complete: 64 of the 65 included studies (98%) reported at least eight of the 10 pre‐specified parameters, and all studies reported replication, water temperature, initial body weight, and feeding frequency. Ammonia concentration was the least consistently reported parameter (47/65, 72%). Sixty‐three studies (97%) used at least three replicates per treatment, with three replicates being the most common design (52 studies); two studies used only two replicates. Twenty‐nine studies (45%) additionally incorporated a pathogen or stress challenge (Supporting Information 2: Table S1).

A total of 1174 individual dose–control comparisons were extracted from the 65 included studies. After excluding comparisons with missing data, 1161 comparisons were used for the dose–response meta‐regression analyses. For the forest plots (Figures 3–11), comparisons were first aggregated at the biomarker level within each outcome category, yielding 123 pooled rows in total. The *k* values shown in the forest plots therefore refer to these aggregated biomarker‐level estimates rather than to individual comparisons, which explains why they do not sum to the total number of extracted effect sizes.

**Figure 3:**
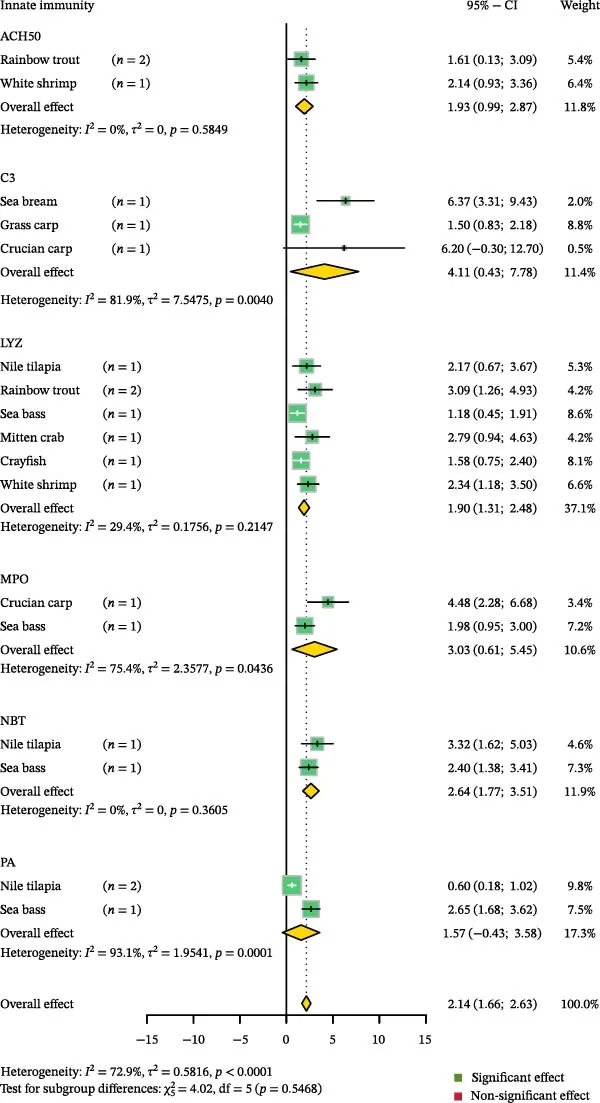
Forest plot showing pooled effect sizes (*g*) for innate immune biomarkers in butyrate‐supplemented finfish and crustaceans.

**Figure 4:**
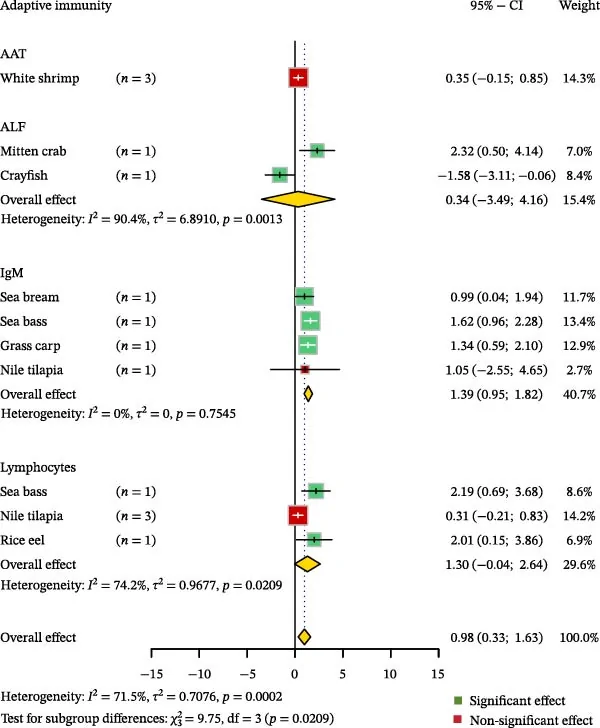
Forest plot showing pooled effect sizes for adaptive immune biomarkers in butyrate‐supplemented finfish and crustaceans.

**Figure 5:**
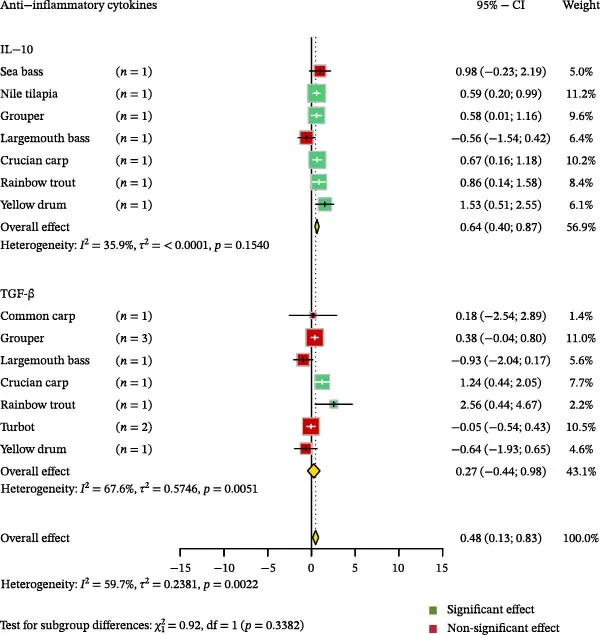
Forest plot showing pooled effect sizes for anti‐inflammatory cytokines following dietary butyrate supplementation.

**Figure 6:**
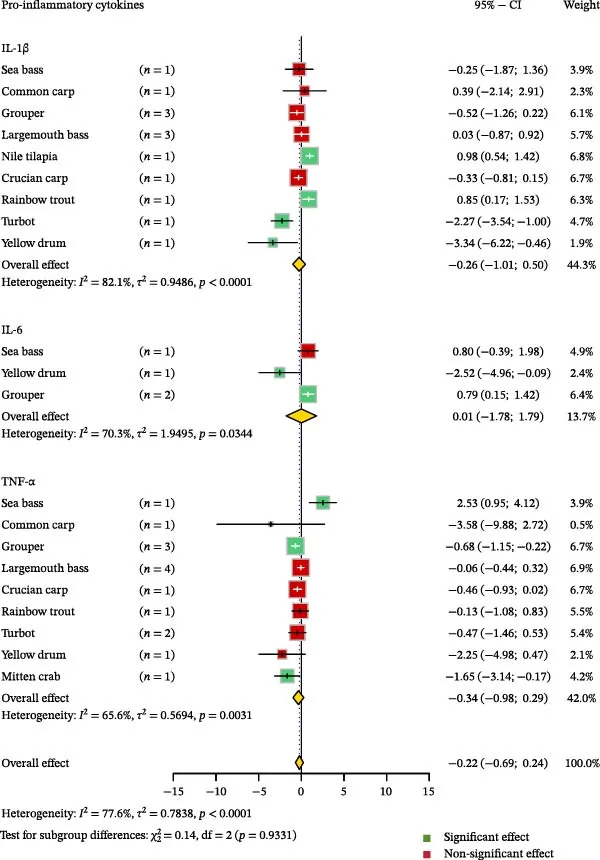
Forest plot showing pooled effect sizes for pro‐inflammatory cytokines following dietary butyrate supplementation.

**Figure 7:**
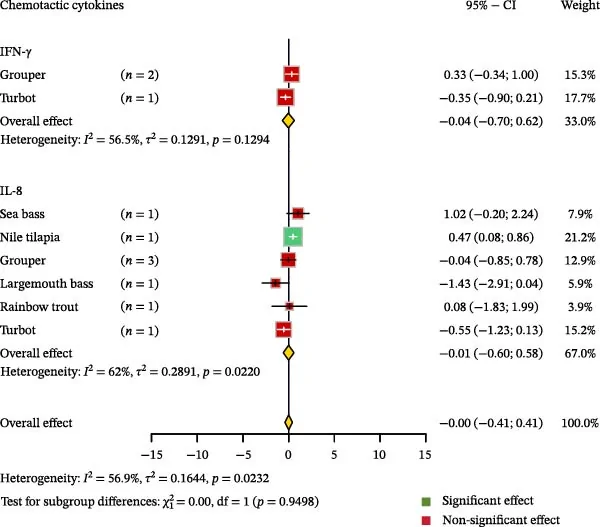
Forest plot showing pooled effect sizes for chemotactic cytokines in butyrate‐supplemented finfish and crustaceans.

**Figure 8:**
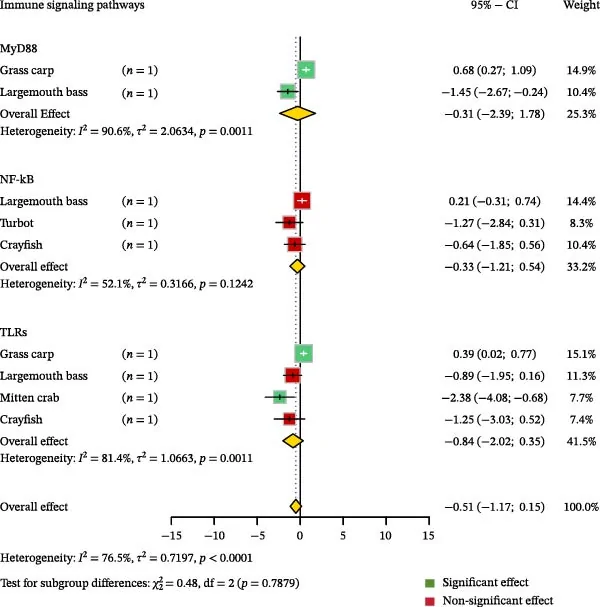
Forest plot showing pooled effect sizes for immune signaling molecules involved in pathogen recognition and inflammatory regulation.

**Figure 9:**
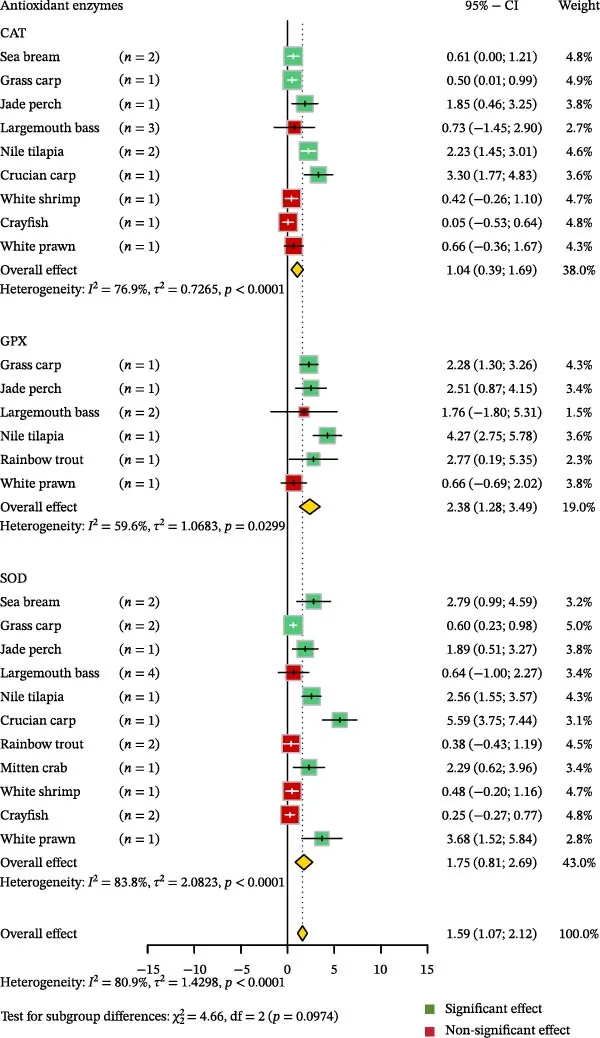
Forest plot showing pooled effect sizes for antioxidant enzyme activities in butyrate‐supplemented finfish and crustaceans.

**Figure 10:**
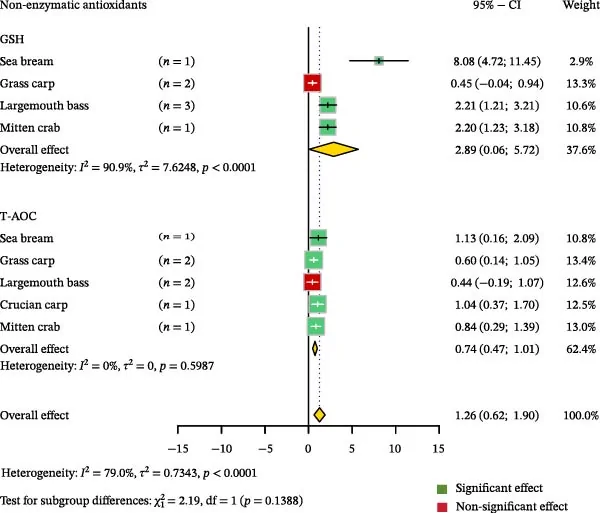
Forest plot showing pooled effect sizes for nonenzymatic antioxidant indicators in butyrate‐supplemented finfish and crustaceans.

**Figure 11:**
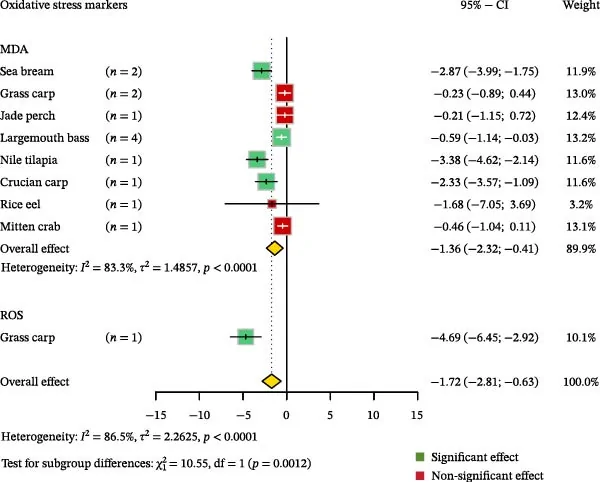
Forest plot showing pooled effect sizes for oxidative stress biomarkers in butyrate‐supplemented finfish and crustaceans.

### 3.2. Immunomodulatory Efficacy of Dietary Butyrate

Dietary butyrate supplementation generally enhanced innate immune responses across both finfish and crustaceans. The pooled meta‐analytic estimate revealed a large effect size (*g* = 2.14, 95% CI: 1.66–2.63; *p*  < 0.001; *k* = 17) for innate immune markers, including lysozyme activity, complement components, respiratory burst activity, and phagocytic responses (Figure 3). These biomarkers showed no significant differences (*p* = 0.547), although this test may have had limited power. Despite substantial heterogeneity among studies (*I* ^2^ = 72.9%), the direction of effects remained generally positive across species and experimental designs.

Adaptive immune responses also improved following dietary butyrate supplementation, although the effects were more moderate and biomarker‐specific. The overall effect size for adaptive immune responses was smaller (*g* = 0.98, 95% CI: 0.33–1.63; *k* = 10; Figure 4). Among adaptive markers, immunoglobulin M (IgM) exhibited the most consistent response (*g* = 1.39; *I* ^2^ = 0.0%), whereas antimicrobial peptides (ALF) showed opposite effects in the two species reporting them (mitten crab *g* = 2.32; crayfish *g* = − 1.58), and lymphocyte indices varied across studies (subgroup differences *p* = 0.021). Only one study reported antiprotease activity (AAT), so consistency could not be assessed.

### 3.3. Cytokine Modulation and Immune Signaling Integration

Dietary butyrate supplementation significantly increased IL‐10 levels (*g* = 0.64; 95% CI: 0.40–0.87; *k* = 7), with low‐to‐moderate heterogeneity (*I* ^2^ = 35.9%; *p* = 0.154 for heterogeneity). In contrast, TGF‐*β* did not show a significant overall change (*g* = 0.27; 95% CI: −0.44–0.98; *k* = 7). The pooled effect for anti‐inflammatory cytokines overall was significant (*g* = 0.48; 95% CI: 0.13–0.83), driven primarily by the consistent increase in IL‐10 (Figure 5).

By contrast, the overall effect for pro‐inflammatory cytokines was not significant (*g* = −0.22; 95% CI: −0.69–0.24; *k* = 21; Figure 6). Within this category, TNF‐*α* also showed no significant overall change (*g* = − 0.34; 95% CI: −0.98–0.29). Although several studies observed a modest reduction in TNF‐*α* levels, the effect was not consistently significant.

Similarly, chemotactic cytokines such as interleukin‐8 (IL‐8) and interferon‐γ (IFN‐γ) (Figure 7) did not show significant pooled effects across studies (overall effect: *g* = −0.00; 95% CI: −0.41–0.41; *I* ^2^ = 56.9%). IFN‐γ showed *g* = −0.04 (95% CI: −0.70–0.62), and IL‐8 showed *g* = −0.01 (95% CI: −0.60–0.58). Upstream immune signaling pathways, including NF‐κB, Toll‐like receptors (TLRs), and MyD88 (Figure 8), also did not show significant pooled effects. These pathways exhibited moderate to high heterogeneity (*I* ^2^ = 52%–91%).

### 3.4. Antioxidant Responses and Oxidative Stress Mitigation

Dietary butyrate supplementation significantly increased antioxidant enzyme activity (*g* = 1.59, 95% CI: 1.07–2.12; *p*  < 0.001; *k* = 26; Figure 9). Among these enzymes, GPx showed the numerically largest effect (*g* = 2.38), followed by superoxide dismutase (SOD; *g* = 1.75) and catalase (CAT; *g* = 1.04), although the differences among enzymes were not significant (*p* = 0.097).

Nonenzymatic antioxidant defenses also showed a significant enhancement (overall *g* = 1.26, 95% CI: 0.62–1.90; *p*  < 0.001; *k* = 9), with total antioxidant capacity (T‐AOC) showing consistent effects across species (*I* ^2^ = 0%; Figure 10).

Furthermore, butyrate supplementation significantly reduced oxidative stress biomarkers (overall *g* = −1.72, 95% CI: −2.81–−0.63; *p* = 0.002; *k* = 9). Within this category, MDA showed *g* = −1.36 (95% CI: −2.32–−0.41) and ROS showed *g* = −4.69 (95% CI: −6.45–−2.92), the latter derived from a single study (Figure 11). Despite substantial heterogeneity among studies (*I* ^2^ = 79%–87%), the direction of effects remained consistently favorable across taxa.

### 3.5. Comparison Across Outcome Categories

Table 1 summarizes the pooled estimates for the nine outcome categories. In absolute magnitude, the largest pooled effects were observed for innate immunity (*g* = 2.14), oxidative stress markers (*g* = −1.72), antioxidant enzymes (*g* = 1.59) and nonenzymatic antioxidants (*g* = 1.26), followed by adaptive immunity (*g* = 0.98). Immune signaling pathways (*g* = −0.51) and anti‐inflammatory cytokines (*g* = 0.48) showed smaller effects, and pro‐inflammatory cytokines (*g* = −0.22) and chemotactic cytokines (*g* = −0.00) showed the smallest. Pooled effects were not significant for pro‐inflammatory cytokines, chemotactic cytokines, and immune signaling pathways. These magnitudes were not compared statistically and are not strictly comparable, because the categories differ in biomarker type, tissue, and heterogeneity, and the oxidative stress estimate depends largely on MDA and on a single ROS study.

**Table 1:** Comparison of standard random‐effects, multilevel, and robust variance estimation models for each outcome category.

Outcome	*k*	Standard *g* (95% CI)	Multilevel *g* (95% CI)	Multilevel *p*	RVE *p*
Innate immunity	17	2.14 [1.66–2.63]	2.15 [1.45–2.86]	<0.0001	<0.001
Adaptive immunity	10	0.98 [0.33–1.63]	0.89 [0.18–1.59]	0.014	0.045
Pro‐inflammatory cytokines	21	−0.22 [−0.69–0.24]	−0.25 [−0.90–0.40]	0.443	0.461
Chemotactic cytokines	8	−0.00 [−0.41–0.41]	0.05 [−0.47–0.57]	0.850	0.857
Anti‐inflammatory cytokines	14	0.48 [0.13–0.83]	0.45 [0.08–0.82]	0.016	0.048
Immune signaling pathways	9	−0.51 [−1.17–0.15]	−0.60 [−1.50–0.31]	0.197	0.268
Antioxidant enzymes	26	1.59 [1.07–2.12]	1.36 [0.67–2.06]	0.0001	0.0033
Nonenzymatic antioxidants	9	1.26 [0.62–1.90]	0.96 [0.62–1.31]	<0.0001	0.0087
Oxidative stress markers	9	−1.72 [−2.81 to −0.63]	−1.44 [−2.34 to −0.53]	0.0019	0.019

Abbreviations: CI, confidence interval; *g*, Hedges’ *g*; *k*, number of effect sizes; RVE, robust variance estimation.

### 3.6. Dose–response Relationships

Meta‐regression with dietary butyrate inclusion level as a continuous moderator identified significant dose–response relationships for innate immunity (*β* = +0.717, 95% CI: 0.207–1.227; Q_model(1) = 7.58, *p* = 0.0059), immune signaling pathways (*β* = −0.453, 95% CI: −0.797 to −0.108; Q_model(1) = 6.64, *p* = 0.010), and oxidative stress markers (*β* = + 0.647, 95% CI: 0.054–1.240; Q_model(1) = 4.57, *p* = 0.033). Marginal trends were observed for anti‐inflammatory cytokines (*p* = 0.066), antioxidant enzymes (*p* = 0.084), and adaptive immunity (*p* = 0.094). Pseudo‐*R* ^2^ values were low throughout (0%–6.33%), indicating that the dietary inclusion level accounted for only a small fraction of the residual heterogeneity; residual heterogeneity remained high in all models (*I* ^2^ = 50.4%–86.4%). Dose therefore appears to be one contributing moderator rather than the principal driver of variability across studies (Table 2).

**Table 2:** Dose–response meta‐regression results by outcome category.

Outcome	*k*	Slope *β* [95% CI]	SE	*p*‐Value	Pseudo‐*R* ^2^	Q_model (df = 1)	*p* (Q_model)
Innate immunity	192	+0.717 [0.207; 1.227]	0.260	0.0059	2.25%	7.58	0.0059
Adaptive immunity	96	−0.269 [−0.583; 0.045]	0.160	0.0935	1.71%	2.81	0.0935
Pro‐inflammatory cytokines	199	−0.017 [−0.619; 0.585]	0.307	0.9556	0.00%	0.003	0.9556
Chemotactic cytokines	74	−0.874 [−3.690; 1.941]	1.436	0.5427	0.00%	0.37	0.5427
Anti‐inflammatory cytokines	120	+1.774 [−0.114; 3.662]	0.963	0.0655	1.63%	3.39	0.0655
Immune signaling pathways	82	−0.453 [−0.797; −0.108]	0.176	0.0100	4.15%	6.64	0.0100
Antioxidant enzymes	224	+0.400 [−0.054; 0.854]	0.232	0.0842	0.05%	2.98	0.0842
Nonenzymatic antioxidants	94	−0.009 [−0.263; 0.244]	0.129	0.9430	0.00%	0.005	0.9430
Oxidative stress markers	80	+0.647 [0.054; 1.240]	0.303	0.0325	6.33%	4.57	0.0325

Abbreviations: *β*, slope coefficient; CI, confidence interval; *k*, number of effect sizes; Pseudo‐*R* ^2^, proportion of between‐study heterogeneity accounted for by dose; Q_model, omnibus test of moderators; SE, standard error.

### 3.7. Sensitivity Analyses

Sensitivity analyses accounting for the nonindependence of effect sizes using multilevel random‐effects models with species as the clustering variable and RVE yielded results consistent with the standard random‐effects models across all outcome categories (Table 1). For innate immunity, the multilevel model yielded *g* = 2.15 (95% CI: 1.45–2.86, *p*  < 0.0001), and the RVE yielded *g* = 2.15 (*p*  < 0.001), compared with *g* = 2.14 (95% CI: 1.66–2.63) from the standard model. For adaptive immunity, the multilevel model yielded *g* = 0.89 (95% CI: 0.18–1.59, *p* = 0.014), and the RVE yielded *g* = 0.89 (*p* = 0.045). For antioxidant enzymes, the multilevel model yielded *g* = 1.36 (95% CI: 0.67–2.06, *p* = 0.0001), and the RVE yielded *g* = 1.36 (*p* = 0.0033). For oxidative stress markers, the multilevel model yielded g = −1.44 (95% CI: −2.34 to −0.53, *p* = 0.0019), and the RVE yielded *g* = −1.44 (*p* = 0.019). Across all outcomes, the direction and statistical significance of the effects were maintained.

We conducted a second sensitivity analysis excluding the three most frequently studied species (largemouth bass, Pacific white shrimp, and Nile tilapia) to determine whether these well‐represented systems drove the pooled effects (Table 3). Across all nine outcome categories, the direction of the pooled effect was maintained, and changes in magnitude were small (|Δ*g*| ≤ 0.27). The largest change was observed for adaptive immunity (*g* = 0.98 to *g* = 1.25), where the effect became slightly stronger after exclusion. For the two largest outcome categories, antioxidant enzymes (Δ*g* = 0.003) and innate immunity (Δ*g* = 0.07), the estimates were essentially unchanged. These results indicate that the observed benefits of dietary butyrate are not attributable solely to the three dominant species.

**Table 3:** Sensitivity analysis excluding the three dominant species (largemouth bass, Pacific white shrimp, and Nile tilapia).

Outcome	*k* (full)	*g* (full) [95% CI]	*k* (excl.)	*g* (excl.) [95% CI]	Δ*g*
Innate immunity	17	2.14 [1.66; 2.63]	12	2.22 [1.68; 2.76]	+0.07
Adaptive immunity	10	0.98 [0.33; 1.63]	7	1.25 [0.37; 2.13]	+0.27
Pro‐inflammatory cytokines	21	−0.22 [−0.69; 0.24]	18	−0.38 [−0.94; 0.18]	−0.15
Chemotactic cytokines	8	−0.001 [−0.41; 0.41]	6	−0.05 [−0.45; 0.36]	−0.05
Anti‐inflammatory cytokines	14	0.48 [0.13; 0.83]	11	0.63 [0.29; 0.97]	+0.15
Immune signaling pathways	9	−0.51 [−1.17; 0.15]	6	−0.51 [−1.45; 0.42]	−0.004
Antioxidant enzymes	26	1.59 [1.07; 2.12]	18	1.60 [0.95; 2.24]	+0.003
Nonenzymatic antioxidants	9	1.26 [0.62; 1.90]	7	1.44 [0.42; 2.46]	+0.18
Oxidative stress markers	9	−1.72 [−2.81; −0.63]	7	−1.67 [−2.98; −0.37]	+0.05

Abbreviations: CI, confidence interval; Δ*g*, change in pooled effect after exclusion; *g*, Hedges’ *g*; *k*, number of effect sizes.

### 3.8. Publication Bias and Trim‐and‐Fill

Publication‐bias diagnostics indicated significant funnel‐plot asymmetry for innate immunity (Egger’s test *p*  < 0.001; Begg’s test *p* = 0.0051) and for antioxidant enzymes (Egger’s test *p* = 0.0001; Begg’s test *p* = 0.0022), whereas no significant asymmetry was detected for adaptive immunity, pro‐inflammatory cytokines, or anti‐inflammatory cytokines (all *p*  > 0.24). For the remaining four outcome categories, *k* < 10 and formal asymmetry testing was not performed, as both tests are underpowered below this threshold.

Trim‐and‐fill adjustment was applied to all outcome categories (Table 4). For innate immunity, we imputed nine potentially missing studies, and the pooled estimate decreased from *g* = 2.14 (95% CI: 1.66–2.63) to *g* = 1.33 (95% CI: 0.63–2.02; *p* = 0.0002). For antioxidant enzymes, eight studies were included, and the estimate fell from *g* = 1.59 (95% CI: 1.07–2.12) to *g* = 0.84 (95% CI: 0.16–1.52; *p* = 0.0154). In both cases, the direction and statistical significance of the effect were preserved, although the magnitude was substantially attenuated. For nonenzymatic antioxidants and oxidative stress markers, adjustment rendered the pooled effects nonsignificant (*p* = 0.314 and *p* = 0.335, respectively), so these results should be interpreted with particular caution.

**Table 4:** Publication bias diagnostics and trim‐and‐fill adjusted estimates.

Outcome	*k*	Egger *p*	Begg *p*	*k*_0_ imputed	Adjusted *g* [95% CI]	Adjusted *p*
Innate immunity	17	<0.001	0.0051	9	1.33 [0.63; 2.02]	0.0002
Adaptive immunity	10	0.3868	0.9287	2	0.74 [0.08; 1.40]	0.0291
Pro‐inflammatory cytokines	21	0.2493	0.3339	4	0.02 [−0.51; 0.55]	0.9360
Chemotactic cytokines	8	n.c. (*k* <10)	n.c. (*k* <10)	1	0.10 [−0.35; 0.54]	0.6645
Anti‐inflammatory cytokines	14	0.8516	0.5470	1	0.43 [0.07; 0.79]	0.0194
Immune signaling pathways	9	n.c. (*k* <10)	n.c. (*k* <10)	4	0.19 [−0.66; 1.04]	0.6599
Antioxidant enzymes	26	0.0001	0.0022	8	0.84 [0.16; 1.52]	0.0154
Nonenzymatic antioxidants	9	n.c. (*k* <10)	n.c. (*k* <10)	3	0.71 [−0.67; 2.10]	0.3138
Oxidative stress markers	9	n.c. (*k* <10)	n.c. (*k* <10)	3	−0.68 [−2.06; 0.70]	0.3348

Abbreviations: CI, confidence interval; *g*, Hedges’ *g*; *k*, number of effect sizes; n.c., not computed (Egger’s and Begg’s tests were not performed for subgroups with *k* < 10).

## 4. Discussion

### 4.1. Synthesis of Principal Findings

This meta‐analysis synthesizes the effects of dietary butyrate supplementation on immune and antioxidant responses across finfish and crustaceans. Overall, the results indicate that butyrate generally enhances host physiological defenses, consistent with previous narrative reviews on short‐chain fatty acids in aquaculture [22–25]. The largest pooled effects were observed for innate immunity (*g* = 2.14) and oxidative stress markers (g = − 1.72), followed by antioxidant enzymes (*g* = 1.59), nonenzymatic antioxidants (*g* = 1.26), and adaptive immunity (*g* = 0.98). Pooled effects on pro‐inflammatory cytokines, chemotactic cytokines, and immune signaling pathways were not significant, in line with the variable responses reported in individual studies [32, 33, 36].

In dose–response meta‐regression, the dietary inclusion level explained only a small fraction of the heterogeneity (pseudo‐*R* ^2^ ≤ 6.3%), and no association remained significant after correction for multiple testing. Dose therefore appears to be one contributing moderator rather than the principal driver of variability across studies. Because we did not retain a study‐level identifier, we could not formally test species, life stage, habitat, or butyrate form as moderators. The variability in effect sizes likely reflects biological and methodological differences among trials, as suggested for other feed additives in aquaculture [42, 60].

To our knowledge, this is the first meta‐analysis of dietary butyrate across immune and antioxidant endpoints in aquaculture species. It identifies where the evidence is relatively strong (innate immunity, antioxidant enzymes), weaker (adaptive immunity, IL‐10, MDA), or absent (pro‐inflammatory, chemotactic, and signaling markers), and provides a basis for future dose–response and long‐term trials.

### 4.2. Innate vs. Adaptive Immunomodulation

The present meta‐analysis reveals a larger pooled effect for innate than for adaptive immune markers in butyrate‐fed aquatic animals. Across studies, butyrate supplementation significantly increased key innate immune biomarkers, including lysozyme activity, complement components, and phagocytic capacity [33, 35, 36]. These results are consistent with butyrate’s role as a histone deacetylase (HDAC) inhibitor, which promotes transcription of antimicrobial peptides and innate immune effectors while modulating transcription factors that coordinate early host defense responses [28, 61].

From an applied perspective, this stimulation of innate immunity may be particularly relevant in aquaculture systems, where early pathogen elimination is critical for preventing disease outbreaks. Enhanced lysozyme activity and complement responses have been associated with increased survival following bacterial challenge in fish and crustaceans, suggesting, without demonstrating, that the observed increases may be relevant for disease resistance [32, 62].

In contrast, adaptive immune responses showed more moderate and selective improvements. Among the evaluated biomarkers, IgM showed the most consistent increase, suggesting that butyrate may support humoral immune responses [34, 39]; however, the included studies did not measure B‐cell maturation or antibody production. This enhancement did not extend uniformly across other adaptive parameters, indicating that butyrate may exert a targeted rather than generalized influence on acquired immunity.

This selective response is biologically plausible. In mammals, butyrate has been shown to promote differentiation of regulatory T cells through HDAC inhibition [63–65], potentially balancing immune activation with regulatory control; whether this occurs in fish is unknown. Such dual regulation, if present in aquatic species, may prevent excessive immune stimulation while maintaining effective pathogen defense.

Overall, these findings suggest that butyrate may strengthen innate defenses, with a smaller and less consistent effect on adaptive responses. This profile may be relevant in intensive aquaculture production systems, where early immune protection and controlled long‐term immunity are essential for maintaining health and production stability.

### 4.3. Cytokine Regulation and Immune Signaling

The cytokine responses observed in this meta‐analysis indicate that dietary butyrate helps establish a balanced immune response in aquatic organisms. Rather than broadly stimulating immune activity, butyrate appears to fine‐tune inflammatory signaling, promoting immune regulation while preserving pathogen defense capacity.

Across studies, pro‐inflammatory cytokines such as IL‐1β and IL‐6 generally remained unchanged, whereas TNF‐*α* tended to decrease in some studies, although the pooled effect was not statistically significant [62, 66]. In contrast, the anti‐inflammatory cytokine IL‐10 was reliably upregulated [35, 67], suggesting the activation of immune‐resolution pathways that limit chronic inflammation while maintaining immune responsiveness. This pattern aligns with established models of immune regulation in fish and crustaceans, where controlled attenuation of inflammatory mediators prevents tissue damage without compromising host defense [68].

Mechanistically, these patterns are consistent with butyrate’s influence on inflammatory signaling pathways. Butyrate inhibits HDAC activity, thereby modifying chromatin accessibility at inflammatory gene loci [28]. This process can reduce IκB kinase phosphorylation and limit NF‐κB nuclear translocation, thereby suppressing the transcription of pro‐inflammatory cytokines such as TNF‐*α* and IL‐1*β* [61, 64]. In parallel, activation of PPAR‐*γ* signaling has been associated with increased IL‐10 production and suppression of inflammatory cascades in mammalian cells [69, 70].

Pooled effects on upstream signaling molecules such as MyD88, TLRs, and NF‐κB subunits were not significant (*g* = −0.51, 95% CI: −1.17–0.15), indicating that the present data do not support a consistent reduction in inflammatory signaling intensity at the pathway level [34, 71].

Some studies have reported anti‐inflammatory responses under challenge conditions [32, 62], suggesting that butyrate may enhance aquatic organisms’ ability to transition from inflammatory activation to immune resolution. This capacity may be relevant in intensive aquaculture production systems, where repeated pathogen exposure can otherwise lead to chronic inflammatory stress [20].

### 4.4. Antioxidant Responses and Oxidative Stress Mitigation

Among the evaluated biological responses, dietary butyrate’s antioxidant effects were among the most consistent. This meta‐analysis indicated that butyrate supplementation increases the activity of major antioxidant enzymes, including superoxide dismutase (SOD), catalase (CAT), and GPx [36, 40]. GPx showed the largest numerical effect (*g* = 2.38), followed by SOD (*g* = 1.75) and CAT (*g* = 1.04), although differences among enzymes were not significant (*p* = 0.097). This coordinated enzymatic response may provide a multilayered defense against ROS, enabling detoxification at multiple stages of oxidative propagation. The GPx response is particularly noteworthy because this enzyme plays a central role in reducing hydrogen peroxide and lipid hydroperoxides, thereby preventing membrane lipid peroxidation and cellular damage [72, 73].

These enzymatic responses were accompanied by increases in nonenzymatic antioxidant defenses, particularly total antioxidant capacity (T‐AOC; *g* = 0.74, *I* ^2^ = 0%) [39, 44], whereas the GSH estimate was imprecise and heterogeneous (*g* = 2.89, *I* ^2^ = 90.9%). Together, these changes suggest a broad reinforcement of the cellular antioxidant defense system rather than isolated alterations in individual biomarkers. Such integrated improvements may be especially important in fish and crustaceans, where an oxidative imbalance can rapidly impair immune competence, metabolic performance, and growth [22, 74].

Mechanistically, these antioxidant effects are consistent with butyrate’s ability to modulate the Keap1–Nrf2 signaling pathway, which regulates the transcription of numerous antioxidant genes [75, 76]. Activation of Nrf2 promotes the expression of enzymes involved in ROS detoxification and GSH synthesis. In addition, butyrate can act as a preferred mitochondrial substrate, improving oxidative metabolism and reducing electron leakage from the respiratory chain [77, 78]. These mechanisms are plausible based on evidence from mammalian and other model systems; however, they should be regarded as potential rather than established in aquatic species, as the included aquaculture studies did not directly measure these pathways.

These molecular and biochemical responses translate into measurable reductions in oxidative damage, as indicated by decreases in MDA (*g* = −1.36, 95% CI: −2.32 to −0.41) [39, 40]. The ROS estimate (*g* = −4.69, 95% CI: −6.45 to −2.92) was derived from a single study (grass carp) and should not be generalized. The particularly strong suppression of ROS in that study suggests that butyrate may reduce oxidative pressure upstream, limiting the initiation of lipid peroxidation cascades, but this hypothesis requires confirmation in additional species [79].

From a practical perspective, this antioxidant conditioning may have implications for aquaculture production. Intensive farming exposes aquatic organisms to multiple stressors, including high stocking densities, handling, and fluctuating environmental conditions, all of which elevate oxidative stress. By strengthening antioxidant defenses and reducing the cellular oxidative burden, butyrate may enhance physiological resilience and help maintain metabolic and immune stability under production stress.

### 4.5. Dose Considerations

In the present meta‐analysis, dietary inclusion level explained only a small fraction of the between‐trial variability (pseudo‐*R* ^2^ ≤6.3%), and no association remained significant after multiple‐testing correction. The nominally significant slopes were positive for innate immunity (larger effects at higher inclusion levels) and for oxidative stress markers (smaller reductions at higher levels), so that no single inclusion level was favored across outcomes. The present data therefore do not support a general recommendation on the inclusion level.

Dose optima are expected to depend on species, life stage, and butyrate form [42]; however, these factors could not be formally tested as moderators because a study‐level identifier was not retained during data extraction. Dose–response trials with several inclusion levels in the target species, life stage, and production system are needed before making such recommendations.

Several mechanisms have been proposed to explain nonlinear dose responses to butyrate in mammalian systems, including saturation of short‐chain fatty acid receptors (GPR41 and GPR43), excessive HDAC inhibition, feedback regulation of SCFA transporters, and metabolic overload associated with excessive *β*‐oxidation of butyrate [28, 77, 80]. These mechanisms are plausible based on evidence from mammalian and other model systems; however, they should be regarded as potential rather than established in aquatic species, as the included aquaculture studies did not directly measure these pathways or test nonlinear models.

Overall, these findings indicate that butyrate should be applied as a functional feed additive in precision nutrition strategies, with dosing strategies tailored to species biology, developmental stage, and production objectives rather than applied uniformly across aquaculture systems.

### 4.6. Heterogeneity and Sources of Variability

Heterogeneity was substantial in all outcome categories (*I* ^2^ = 57%–87%). The forest plots show that species‐level estimates differed: for antioxidant enzymes (Figure 9), estimates were significant for Nile tilapia, crucian carp, grass carp, jade perch, and sea bream, and nonsignificant for largemouth bass (all three enzymes), and for crayfish and Pacific white shrimp (CAT and SOD). These differences could reflect biological variation, but species are confounded with study, laboratory, country, and experimental conditions, and the analysis could not formally test them.

Sixty‐four of the 65 studies (98%) used juveniles or other pre‐adult stages, and only one used adults, so the results cannot be extended to adult animals. Butyrate forms also differed among studies; protected formulations are expected to reach the distal intestine more effectively [23], but the effect of form on the pooled estimates was not tested.

### 4.7. Limitations and Future Research Directions

While this meta‐analysis provides quantitative evidence on the effects of dietary butyrate supplementation in aquaculture species, limitations should be noted. These fall into two broad categories: methodological constraints inherent to the present synthesis and limitations of the available literature.

A study‐level identifier was not retained during data extraction, and dependency among effect sizes was therefore modeled at the species rather than the study level. Dose was the only moderator that could be formally tested; habitat, life stage, butyrate form, and supplementation duration could not be examined as moderators. For the 11 species represented by a single study, species‐level clustering is equivalent to study‐level clustering, and for multistudy species it yields wider confidence intervals than study‐level clustering would. Nonetheless, residual dependency among effect sizes originating from the same experimental unit cannot be fully excluded. In addition, although reporting of experimental conditions was generally complete, most included studies did not describe randomization procedures, allocation concealment, or blinding of outcome assessment, precluding a formal risk‐of‐bias appraisal using instruments such as SYRCLE. Performance and detection bias can therefore not be excluded. Publication‐bias diagnostics indicated funnel‐plot asymmetry for innate immunity and antioxidant enzymes, and trim‐and‐fill adjustment substantially attenuated both pooled estimates (*g* = 2.14 → 1.33 and 1.59 → 0.84) and rendered nonenzymatic antioxidants and oxidative stress markers nonsignificant. These findings suggest that small‐study effects may inflate the apparent magnitude of butyrate’s benefits, and the pooled estimates reported here may be overestimated. Finally, most trials used three replicates per treatment, and several standardized effects exceeded |*g*| = 3; with such small samples, standard deviations are imprecise and standardized mean differences may be inflated.

The evidence base remains geographically and taxonomically unbalanced, with most studies focusing on a limited number of freshwater species within Asian aquaculture systems (72% Asia; 62% China; Figure 2). This concentration makes it difficult to generalize results to other taxa, particularly cold‐water marine species, such as Atlantic salmon. Only one study used adult animals; the results therefore apply primarily to the juvenile stages. Furthermore, much of the available research has been conducted under short‐term laboratory conditions, raising questions about the long‐term sustainability of butyrate supplementation throughout complete commercial production cycles. The pooled analyses also combined different butyrate forms, which differ in gastric stability, intestinal release site, and bioavailability; the number of studies per individual formulation precluded a formal subgroup analysis. Similarly, several studies combined butyrate with other additives, and the effect of butyrate alone could not be isolated. Finally, our understanding of butyrate’s mechanistic action in fish is still evolving, with many studies relying on analogies with mammalian systems, while the specific receptors, signaling pathways, and host–microbiome interactions in aquatic species remain underexplored.

To address these gaps, future research should prioritize (1) dose–response trials with several inclusion levels and adequate replication in the target species and life stages; (2) reporting of study‐level identifiers and raw data to allow multilevel synthesis and formal moderator analyses; (3) mechanistic studies in fish using advanced omics techniques; (4) long‐term trials that assess physiological, zootechnical, and economic performance under commercial conditions; and (5) studies of butyrate combined with other functional feed additives and of broodstock and early life stages. By addressing these priorities, butyrate could evolve from a promising additive into a well‐defined nutritional tool that enhances resilience, productivity, and sustainability in global aquaculture systems.

## 5. Conclusion

To our knowledge, this meta‐analysis provides the first quantitative synthesis jointly assessing the effects of dietary butyrate supplementation on immune responses, inflammatory regulation, and antioxidant responses across finfish and crustacean species. The results indicate that butyrate generally enhanced innate immune defenses and antioxidant capacity, whereas effects on inflammatory markers were more variable, with no significant overall change in pro‐inflammatory cytokines. Dose was the only moderator that could be formally tested; dietary inclusion level explained little of the variability, and no inclusion range was consistently associated with stronger responses. Publication‐bias adjustment attenuated some pooled estimates, so the magnitude of the reported benefits should be interpreted as indicative rather than precise. These findings support the growing role of butyrate as a functional feed additive in aquafeeds and highlight the importance of precision nutritional strategies tailored to species, life stage, and production conditions. Future research should prioritize long‐term production trials, mechanistic investigations of host–microbiome interactions, and validation in underrepresented marine and high‐value species. Such efforts will be essential for translating experimental evidence into practical feed formulation and feeding strategies that enhance the health, resilience, and sustainability in modern aquaculture systems.

## Author Contributions

**Moussa Gouife:** conceptualization, methodology, software, writing – original draft, writing – review and editing. **Tchouli Noufeu:** data curation, investigation, validation, writing – original draft. **Yubin Chen:** data curation, investigation, writing – original draft. **Xiwen Jia:** formal analysis, visualization, writing – original draft. **Jiajie Xu:** funding acquisition, project administration, resources, supervision, writing – review and editing.

## Funding

This work was financially supported by the National Natural Science Foundation of China (Grant 23FAA01731), the Department of Science and Technology of Zhejiang Province (Grant 2024C03128), the Ningbo Municipal Bureau of Science and Technology (Grant 2023H023), the Ningbo Municipal Talent Office (Grant ZX2023000069), and the K.C. Wong Magna Fund at Ningbo University.

## Ethics Statement

This meta‑analysis used only published data; no ethical approval was required.

## Conflicts of Interest

The authors declare no conflicts of interest.

## Supporting Information

Additional supporting information can be found online in the Supporting Information section.

## Supporting information


**Supporting Information 1** PRISMA 2020 checklist

**Supporting Information 2** Table S1: Summary of the 65 studies included in the meta‐analysis, detailing study origin, aquatic species, experimental design, rearing conditions, butyrate supplementation protocols, and pathogen or stress challenges.

**Supporting Information 3** Table S2: Classification of biomarkers into outcome categories.

## Data Availability

The data supporting the findings of this meta‑analysis were extracted from published studies listed in the Supporting Information. The extracted dataset is available from the corresponding author upon reasonable request.
