# Supplementary material for: Dietary Butyrate as a Functional Feed Additive in Aquaculture: A Systematic Review and Meta‐Analysis of Immunological and Antioxidant Responses With Dose–Response Insights
Source: Aquac Nutr. 2026 Oct 21;2026:1873426. doi: 10.1155/anu/1873426 (PMC13686364; doi:10.1155/anu/1873426)
Supplement: Supplementary file 2 [file ANU-2026-1873426-s003.docx]

**TABLE S2.** Classification of biomarkers into outcome categories.

| **Outcome category** | **Biomarkers included** |
| --- | --- |
| Innate immunity | Lysozyme (LYZ), complement components (C3, C4), respiratory burst (NBT), phagocytosis, myeloperoxidase (MPO), antiprotease (PA), ACH50 |
| Adaptive immunity | Immunoglobulin M (IgM), antimicrobial peptides (ALF), lymphocyte indices, antiprotease activity (AAT) |
| Anti-inflammatory cytokines | Interleukin-10 (IL-10), transforming growth factor-β (TGF-β) |
| Pro-inflammatory cytokines | Interleukin-1β (IL-1β), interleukin-6 (IL-6), tumor necrosis factor-α (TNF-α) |
| Chemotactic cytokines | Interleukin-8 (IL-8), interferon-γ (IFN-γ) |
| Immune signaling pathways | NF-κB, Toll-like receptors (TLRs), MyD88 |
| Antioxidant enzymes | Superoxide dismutase (SOD), catalase (CAT), glutathione peroxidase (GPx) |
| Non-enzymatic antioxidants | Reduced glutathione (GSH), total antioxidant capacity (T-AOC) |
| Oxidative stress markers | Reactive oxygen species (ROS), malondialdehyde (MDA) |

**Légende:** Biomarkers were assigned to outcome categories based on their biological function and the nomenclature used in the source studies. A complete study-by-study mapping is provided in Supplementary Table S1.
